# Suppression of Hepatocellular Carcinoma Progression through FOXM1 and EMT Inhibition via Hydroxygenkwanin-Induced miR-320a Expression

**DOI:** 10.3390/biom10010020

**Published:** 2019-12-21

**Authors:** Li-Fang Chou, Chi-Yuan Chen, Wan-Hua Yang, Chin-Chuan Chen, Junn-Liang Chang, Yann-Lii Leu, Miaw-Jene Liou, Tong-Hong Wang

**Affiliations:** 1 Kidney Research Center, Chang Gung Memorial Hospital, Tao-Yuan 33305, Taiwan; d928209@gmail.com; 2 Tissue Bank, Chang Gung Memorial Hospital, Tao-Yuan 33305, Taiwan; d49417002@gmail.com (C.-Y.C.); chinchuan@mail.cgu.edu.tw (C.-C.C.); 3 Graduate Institute of Health Industry Technology and Research Center for Industry of Human Ecology, College of Human Ecology, Chang Gung University of Science and Technology, Tao-Yuan 33303, Taiwan; 4 Department of Pathology and Laboratory Medicine Taipei Veterans General Hospital, Hsinchu Branch, Hsin-chu 31064, Taiwan; dammy310@yahoo.com.tw; 5 Department of Medical Laboratory Science and Biotechnology, Yuanpei University of Medical Technology, Hsin-chu 30015, Taiwan; 6 Graduate Institute of Natural Products, Chang Gung University, Tao-Yuan 33303, Taiwan; ylleu@mail.cgu.edu.tw; 7 Department of Pathology and Laboratory Medicine, Taoyuan Armed Forces General Hospital, Tao-Yuan 32551, Taiwan; junn9liang@yahoo.com.tw; 8 Biomedical Engineering Department, Ming Chuan University, Tao-Yuan 33348, Taiwan; 9 Chinese Herbal Medicine Research Team, Healthy Aging Research Center, Chang Gung University, Tao-Yuan 33303, Taiwan; 10 Center for Traditional Chinese Medicine, Chang Gung Memorial Hospital, Tao-Yuan 33305, Taiwan; 11 Division of Endocrinology and Metabolism, Department of Internal Medicine, Chang Gung Memorial Hospital, Tao-Yuan 33305, Taiwan; lioumj@cgmh.org.tw; 12 Liver Research Center, Department of Hepato-Gastroenterology, Chang Gung Memorial Hospital, Tao-Yuan 33305, Taiwan

**Keywords:** liver cancer, hydroxygenkwanin, epithelial–mesenchymal transition, FOXM1, miR-320a

## Abstract

*Daphne genkwa*, a Chinese medicinal herb, is used frequently in Southeast Asian countries to treat diseases; the flavonoid hydroxygenkwanin (HGK) is extracted from its flower buds. The bioactivity of HGK, particularly as an anti-liver cancer agent, has not been explored. In this study, human hepatocellular carcinoma (HCC) cell lines and an animal xenograft model were employed to investigate both the activity of HGK against liver cancer and its cellular signaling mechanisms. HCC cells treated with HGK were subjected to cell function assays. Whole transcriptome sequencing was used to identify genes whose expression was influenced by HGK, and the flavonoid’s cancer suppression mechanisms were further investigated through gain- and loss-of-function assays. Finally, in vitro findings were tested in a mouse xenograft model. The data showed that HGK induced the expression of the microRNA miR-320a, which in turn inhibited the expression of the transcription factor ‘forkhead box protein M1’ (FOXM1) and downstream FOXM1-regulated proteins related to epithelial–mesenchymal transition, thereby leading to the suppression of liver cancer cell growth and invasion. Significant inhibition of tumor growth was also observed in HGK-treated mice. Hence, the present study demonstrated the activity of HGK against liver cancer and validated its potential use as a therapeutic agent.

## 1. Introduction

According to the World Health Organization statistics published in 2018, liver cancer is the seventh most common malignancy in the world and is also the second leading cause of cancer-related death. Globally, approximately 780,000 people die from liver cancer every year [1]. Liver cancer is one of the most difficult cancers to treat owing primarily to its drug resistance and high metastatic propensity [2,3]. The causes of liver cancer are also extremely complex, with the main risk factors being chronic hepatitis B and hepatitis C, alcoholic hepatitis, and non-alcoholic fatty liver disease [4,5]. In Africa and Asia, hepatitis B virus infection is the etiologic factor in approximately 60% of patients with liver cancer [6,7]. In addition to triggering the malignant transformation of liver cells during the progression of chronic hepatitis to cirrhosis [8], hepatitis B virus may also cause genetic recombination and the activation of oncogenes such as ‘forkhead box protein M1’ (*FOXM1*) [9]. Hepatitis B virus may also cause tumor suppressor gene inactivation after its viral DNA integrates into the host genome [10,11,12]. These phenomena can induce uncontrolled cell growth and ultimately result in the development of liver cancer. 

Surgical resection is a common intervention for patients with liver cancer [13]. However, patients with advanced metastatic disease typically receive chemotherapy or radiotherapy as the primary mode of treatment [3]. Presently, sorafenib and bevacizumab are the most widely used targeted therapies in liver cancer treatment [14,15]. Sorafenib, a multikinase inhibitor that blocks the activation of kinases associated with the regulation of tumor growth and angiogenesis, has been recognized as the most effective targeted drug against liver cancer [16]; however, it only prolongs patient survival by approximately 3 months [17,18]. Additionally, most drugs that target liver cancer can cause severe side effects that inevitably have a profound negative impact on the patient’s quality of life [19]. Therefore, the development of therapeutic drugs with higher efficacies and milder side effects remains a key imperative of liver cancer research.

The application of Chinese medicinal herbs in the treatment of diseases has a long history in Southeast Asian countries. With advancements in substance extraction and component identification technologies, the effective ingredients of many Chinese medicinal herbs have been successfully purified and their functions identified [20]. Certain Chinese medicinal herbal extracts, such as artemisinin and curcumin, are already being used in cancer treatment and have been proven to both suppress tumor growth and metastasis and prolong patient survival [21,22,23,24,25]. Compared with Western medicine, these natural compounds trigger relatively milder physiological side reactions and are highly suitable for use as adjuvants to other drugs for the enhancement of treatment efficacy.

*Daphne genkwa*, a medicinal herb that is distributed widely throughout Southeast Asia, has long been used as an anti-inflammatory agent as well as for pain relief, sedation, and the treatment of edema and asthma [26,27]. The flower buds of *D. genkwa* contain an abundance of flavonoids and daphne diterpene esters, including the flavonoid hydroxygenkwanin (HGK) [28,29]. A previous study found that HGK could induce mitochondrial injury in brain cancer cells and concurrently trigger DNA breakage and cell cycle arrest, ultimately inducing apoptosis [30,31]. However, research on the bioactivity of HGK has been limited, and there are no published studies on its efficacy against other types of cancer. Therefore, in the present study, human liver cancer cells and an animal model were used to test the efficacy of HGK against liver cancer and to investigate its regulatory mechanisms in detail. The results of this study indicated that HGK could significantly suppress the proliferation, migration and invasion of liver cancer cells. Additionally, it was found that HGK induced the expression of the microRNA (miRNA) miR-320a, which in turn inhibited the expression of the transcription factor FOXM1 and downstream FOXM1-regulated genes that are associated with epithelial–mesenchymal transition (EMT), thereby leading to the suppression of liver cancer cell growth and (potentially) metastasis. Taken together, the data demonstrated that HGK is effective against liver cancer and is of potential use as a therapeutic agent against this disease. 

## 2. Materials and Methods

### 2.1. Cell Lines

The hepatocellular carcinoma cell lines HepG2 and Huh7 were purchased from the American Type Culture Collection (Manassas, VA, USA) and donated by Chau-Ting Yeh of Chang Gung Memorial Hospital. Pan-Chyr Yang of Taiwan University benevolently provided human skin fibroblasts (HFB) used in this study. The cells were cultured in Dulbecco’s modified Eagle medium (DMEM) containing 10% fetal bovine serum at 37 °C in a 5% CO_2_ incubator. 

### 2.2. Drug, Antibodies, Plasmids, and Small Interfering RNA (siRNA)

HGK powder (purity > 99% as verified by high-precision liquid chromatography) was purchased from Shanghai BS Bio-Tech Co., Ltd. (Shanghai, China). Polyclonal antibodies against FOXM1 (#13147-1-AP), E-cadherin (#3195), N-cadherin (#13116), vimentin (#5741), twist (GTX127310), snail (#3879) and β-actin (#8480) were purchased from Proteintech (Rosemont, IL, USA), GeneTex (Irvine, CA, USA) and Cell Signaling Technology (Beverly, MA, USA). Secondary antibodies were purchased from Santa Cruz Biotechnology (Santa Cruz, CA, USA). Prestained protein marker and TOOLSmart RNA extractor were purchased from BIOTOOLS (New Taipei City, Taiwan). The commercialized miR-320a mimic and inhibitor were purchased from Thermo Fisher Scientific (Waltham, MA, USA). Commercialized siRNA targeting *FOXM1* as well as negative control siRNA were purchased from Thermo Fisher Scientific (Waltham, MA, USA). 

### 2.3. Cell Proliferation Assay 

Huh7 or HepG2 cells were seeded at 3 × 10^3^ per well in 96-well E-plates and cultured in DMEM in the presence or absence of various concentrations of HGK. The cell proliferation rates were monitored with an xCELLigence real-time cell analyzer (Roche Life Science, Indianapolis, IN, USA) according to the manufacturer’s instructions.

### 2.4. Cell Migration and Invasion Assays

The wound healing assay was performed as described earlier [32]. Cells were seeded in 6-well plates and cultured to 90% confluence. Cells were scraped with a p200 tip (time 0), and the medium was replaced with low-serum culture medium that contained different concentrations of HGK, or no HGK. Wound area was measured from images (five fields) taken at stipulated times by digital planimetry using the ImageJ software (NIH, Bethesda, MD, USA).

The migration and invasion characteristics of cells were examined using ThinCert Tissue Cell Culture Inserts (Greiner Bio-One, Kremsmunster, Austria) as described earlier [32]. For the migration assay, 5 × 10^4^ cells were resuspended in 100 μL serum-free culture medium (DMEM) that contained or did not contain HGK and placed in the upper chambers. The lower chambers were filled with 500 μL DMEM medium that contained 10% FBS. Twenty-four hours after treatment, the cells were fixed on a membrane using methanol, and cells on the upper surface of the membrane were removed with cotton swabs. The membrane was washed twice with PBS and then stained with 0.1% crystal violet. The stained cells were imaged using Image-Pro version 6.2 software (Media Cybernetics, Rockville, MD, USA). Cell counts were obtained from five random fields at 100× magnification. For the invasion assay, the membrane was coated with 30 mg/cm^2^ Matrigel (ECM gel, Sigma–Aldrich, St. Louis, MO, USA) in order to form a matrix barrier. The procedure for performing the invasion assay was the same as that of a migration assay except that the permeating time for the cells was 48 h.

### 2.5. Gene Expression Profiling 

Huh7 and HepG2 cells were treated with 40 μM and 30 μM HGK for 48 h, respectively. Total RNA from cells was isolated using an RNeasy mini kit (QIAGEN, Gaithersburg, MD, USA) and subjected to whole transcriptome sequencing as described previously [33]. The purity and integrity of RNA from cells were assessed using the Bioanalyzer 2100 system (Agilent Technologies, Santa Clara, CA, USA). Sequencing libraries were constructed using the rRNA-depleted RNA and the NEBNext Ultra Directional RNA Library Prep Kit for Illumina (NEB, Ipswich, MA, USA) according to manufacturer’s instructions. Sequencing was performed using the Illumina Hiseq 2000 platform.

### 2.6. Quantitative Real-Time Reverse Transcription (RT) PCR

Total RNA from cells was isolated using an RNeasy mini kit (QIAGEN), followed by treatment with RQ1 RNase-free DNase (Promega, Madison, WI, USA) according to the manufacturer’s instructions. The treated RNA samples were subjected to RT, and the products were subjected to quantitative real-time PCR to detect *FOXM1* expression using the TaqMan gene expression assay (Applied Biosystems, Foster City, CA, USA); *GAPDH* was used as an internal control. 

MiRNAs were isolated using a miRNeasy Mini Kit (QIAGEN) and converted into cDNA using TaqMan microRNA Reverse Transcription Kit (Thermo Fisher Scientific). The expression of miR-320a was analyzed using the TaqMan microRNA assay (Thermo Fisher Scientific) with RNU6B used as an internal control.

### 2.7. Transfection and Western Blotting Analysis

Huh7 and HepG2 cells were seeded in 6-well plates at a density of 3 × 10^5^ cells/well overnight. The cells were then transfected with 50 nM siRNA or miRNA inhibitor using Lipofectamine RNAiMAX transfection reagent (Invitrogen, Carlsbad, CA, USA) according to the manufacturer’s instructions. Forty-eight hours later, transfected cells were washed twice with phosphate-buffered saline and then lysed in 200 μL of RIPA lysis buffer (BIOTOOLS, new Taipei city, Taiwan) containing protease inhibitors. Proteins from the supernatant (50 μg) were separated on SDS polyacrylamide gels and then western blotted to detect the levels of FOXM1, E-cadherin, N-cadherin, snail, twist, and β-actin. Primary antibodies were used at working dilutions of 1:1000. The immunoreactive bands were visualized using an enhanced chemiluminescence system (NEN Life Science Products, Boston, MA, USA) and developed on X-ray films. The intensity of each band was quantified using ImageQuant 5.2 (GE Healthcare, Piscataway, NJ, USA).

### 2.8. Animals

Six-week-old male nude mice were purchased from the National Laboratory Animal Center (Taipei, Taiwan), and housed under pathogen-free conditions. Ten healthy mice were randomly assigned to treatment and control groups with each group comprising five mice. All animal experiments were performed after obtaining the approval of the Institutional Animal Care and Use Committee (IACUC) of Chang Gung Memorial Hospital (IACUC approval no.: 2018031301, approval date: 6/19/2018); the experiments followed the Guidelines for the Care and Use of Laboratory Animals (National Institutes of Health). 

### 2.9. Xenograft Assays and Drug Administration 

Huh7 cells at a density of 5 × 10^6^ were resuspended in 100 μL of DMEM with 10% fetal bovine serum and subcutaneously implanted into the left and right flank regions of the mice. All the tumors were staged during the week before drug treatment was initiated. At the beginning of the second week, the five mice from each group were intraperitoneally injected with 100 µL of HGK (at a dose of 1 mg/kg of body weight) or an equal volume of the vehicle control dimethyl sulfoxide (DMSO) 3 times per week. Tumor volumes were measured 3 times per week using the following equation: tumor volume = length × width^2^ × 0.5. Tumor size and body weight of different groups were measured and the calculated mean ± standard deviation (SD) values were used to plot tumor growth curve. Differences between means were determined using Student’s *t*-test. *p* < 0.05 was considered statistically significant. The mice were sacrificed at the end of the experiment, and tumors were excised, weighed, and fixed in formalin for further analysis. 

### 2.10. Immunohistochemistry

The tumors that developed in mice were fixed in formalin and embedded in paraffin, and 2 μm-thick consecutive sections were cut and subjected to immunohistochemical staining using a BOND III autostainer (Leica Biosystems, Wetzlar, Germany) as described previously [34].

### 2.11. Statistical Analysis

Real-time PCR original data as well as western blotting and migration assay results were recorded as continuous variables and analyzed using Student’s *t*-test. All statistical analyses were performed using SPSS 16.0 (SPSS Inc., Chicago, IL, USA) and Excel 2007 (Microsoft Inc., Redmond, WA, USA). All statistical tests were two-sided, and the *p*-values of significance were established at <0.05 (*), <0.01 (**), or <0.001 (***).

## 3. Results

### 3.1. HGK Suppresses the Proliferation, Migration, and Invasion of Liver Cancer Cells

**Figure 1:**
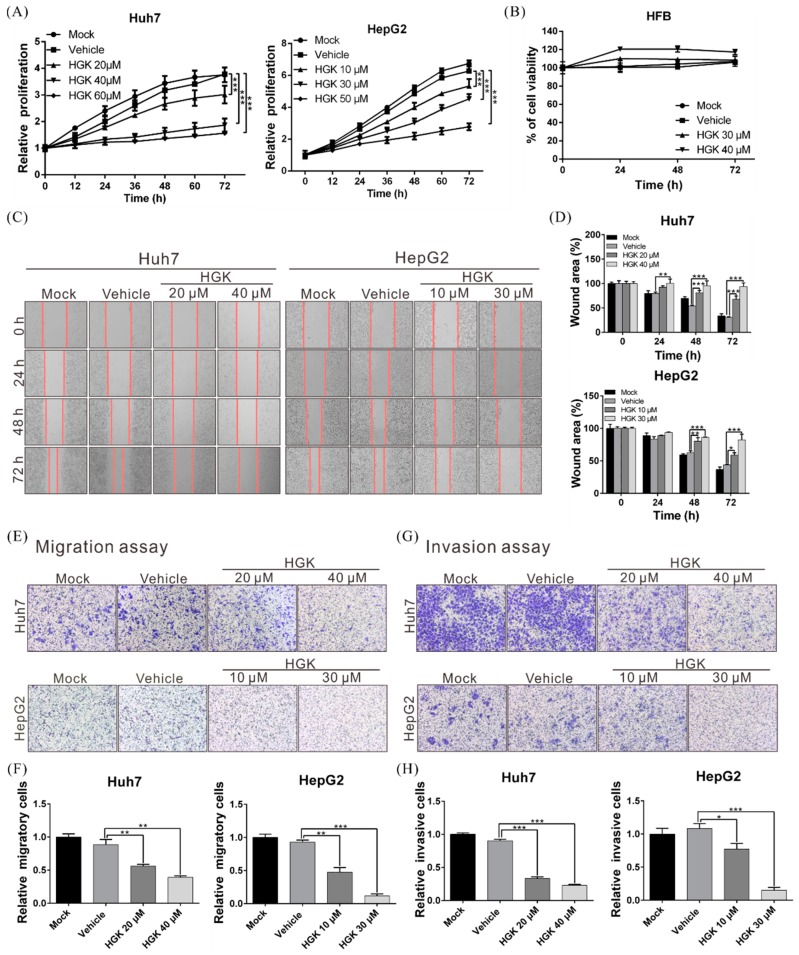
Hydroxygenkwanin (HGK) suppresses the proliferative, migratory, and invasive capacities of liver cancer cells. (**A**) Huh7 and HepG2 cells were treated with various concentrations of HGK and cell proliferation was monitored using the xCELLigence real-time cell analyzer at the indicated time points. (**B**) Human skin fibroblast (HFB) cells were treated with different concentrations of HGK or vehicle (DMSO), and the cell proliferation status was analyzed using the xCELLigence Real-Time Cell Analyzer. (**C**) The migration of Huh7 and HepG2 cells with or without HGK treatment were examined using in vitro wound healing and transwell migration assays. (**E**) quantitative results are shown in (**D**,**F**). (**G**) Hepatocellular carcinoma cell invasion in the presence of absence of HGK treatment was analyzed via transwell assays; the quantitative results are shown in (**H**). * *p* < 0.05, ** *p* < 0.01, and *** *p* < 0.001. All data are expressed as the means ± standard deviations of 3 independent experiments.

The human liver cancer cell lines HepG2 and Huh7 were treated with various concentrations of HGK, following which their proliferation was evaluated using the xCELLigence real-time cell analyzer. HGK significantly suppressed the growth of both cell lines in a dose-dependent manner (Figure 1A). Further analysis revealed that the suppressive effect was significantly greater in the HepG2 cell line, with the half-maximal inhibitory concentrations (IC_50_) of HGK at 27.46 µM and 41.04 µM for HepG2 and Huh7 cells, respectively (data not shown). However, no inhibitory effect was observed on the growth of human skin fibroblast cell line HFB at the above concentrations (Figure 1B). This showed that HGK selectively inhibits the growth of liver cancer cells without significant toxicity to normal cells.

The high mortality rate associated with liver cancer is due to these tumors’ high metastatic propensities. Wound healing and transwell migration assays were thus employed to analyze the influence of HGK on the migratory and invasive phenotypes of the two liver cancer cell lines; both were significantly reduced owing to HGK treatment (Figure 1C–H), indicating that the proliferative and invasive capacities of liver cancer cells can be suppressed by HGK.

### 3.2. HGK Suppresses Tumor Growth in Mice Without Causing Physiological Toxicity

**Figure 2:**
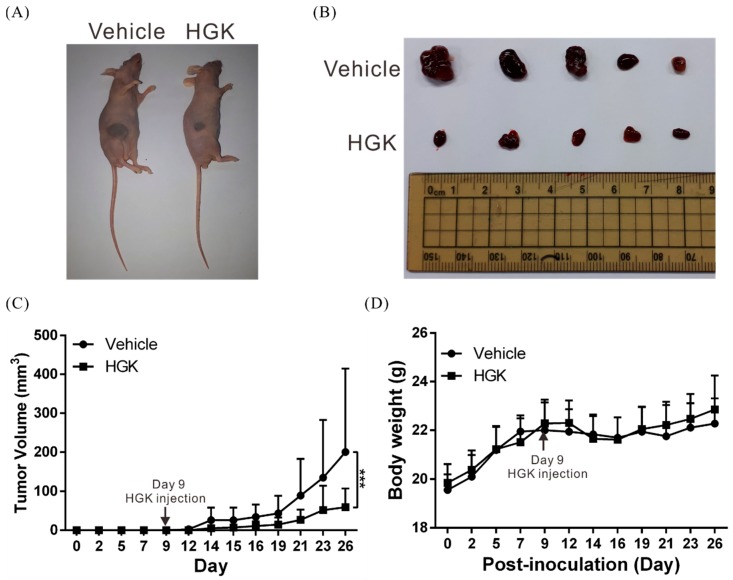
Hydroxygenkwanin (HGK) suppresses tumor growth in mice. (**A**,**B**) 5 × 10^6^ Huh7 cells were injected into the dorsal flanks of nude mice (*N* = 5 from each group) to induce tumor formation. Subsequently, the mice received intraperitoneal injections of HGK (1 mg/kg) or vehicle (DMSO) three times per week. Representative images show the tumor xenografts at 4 weeks after implantation; (**C**) Tumor volumes were measured every 3 days after injection. The volume of each tumor was calculated as follows: length × width^2^ × 0.5. Bars indicate standard deviations; *** *p* < 0.001; (**D**) Body weights were measured every three days after injection, and did not significantly differ between the groups.

A mouse xenograft model was constructed to investigate the anticancer potential of HGK in vivo. Huh7 cells were injected into the dorsal flanks of experimental mice to induce tumor formation, after which the mice received intraperitoneal injections of HGK or vehicle (DMSO) three times per week; tumor growth was measured three times a week. A significant inhibition of tumor growth was observed in mice that received HGK compared with those that received DMSO (Figure 2A–C). Of note, the body weights of the mice in the treated and control groups were not significantly different (Figure 2D). This indicated that HGK exhibited anti-liver cancer activity in vivo without causing significant toxicity to normal tissues.

### 3.3. HGK Inhibits the Expression of the Transcription Factor FOXM1

**Figure 3:**
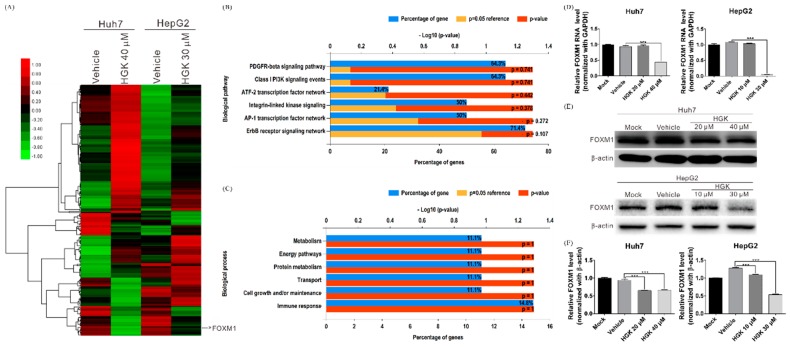
Hydroxygenkwanin (HGK) inhibits *FOXM1* gene expression. (**A**) A heatmap showed the gene expression profile in hepatocellular carcinoma cells treated with or without stated concentrations of HGK for 48 h (red and green indicate higher and lower expression, respectively). Bar charts represent the enriched biological pathways (**B**) and biological processes (**C**) associated with the differentially expressed genes after HGK treatment. (**D**) Quantitative reverse transcription PCR determined the mRNA levels of *FOXM1* after HGK treatment for 48 h; *** *p* < 0.001. (**E**) Western blot analysis showed FOXM1 protein levels after HGK treatment; the quantitative results are shown in (**F**). All data were expressed as the mean ± standard deviation values obtained from three independent experiments. Mock: cells treated with DMEM medium. Vehicle: cells treated with DMSO.

To further elucidate the anticancer mechanisms of HGK, whole transcriptome sequencing was performed to analyze the gene expression patterns in the HGK-treated and -untreated cell lines. The analysis identified 105 genes in HGK-treated cells whose expression levels were at least 2-fold greater or lesser than those in controls (Figure 3A). Kyoto Encyclopedia of Genes and Genomes pathway analysis indicated that these genes largely participated in the regulation of physiological processes such as cell growth, energy metabolism, and immune response (Figure 3B,C). In particular, the expression level of the known oncogene *FOXM1* was significantly decreased after HGK treatment (Figure 3A). Previous studies have indicated that the transcription factor FOXM1 regulates the expression of many other oncogenes; it is also considered an important target for anticancer therapy as it is overexpressed in various malignancies including lung, breast, and liver cancers. To investigate the regulatory effects of HGK on FOXM1, real-time RT-PCR and western blotting were performed to determine FOXM1 mRNA and protein expression, respectively, in the HGK-treated cell lines. Significant decreases in FOXM1 protein levels were observed in both the Huh7 and HepG2 cells after HGK treatment at both low and high doses; however, *FOXM1* mRNA levels only decreased with high HGK doses (Figure 3D–F). This indicates that HGK may influence FOXM1 expression in a multimodal fashion by inhibiting both gene and protein expression through potentially separate mechanisms.

### 3.4. HGK Suppresses the Expression of EMT-Related Genes by Inhibiting FOXM1 Expression

**Figure 4:**
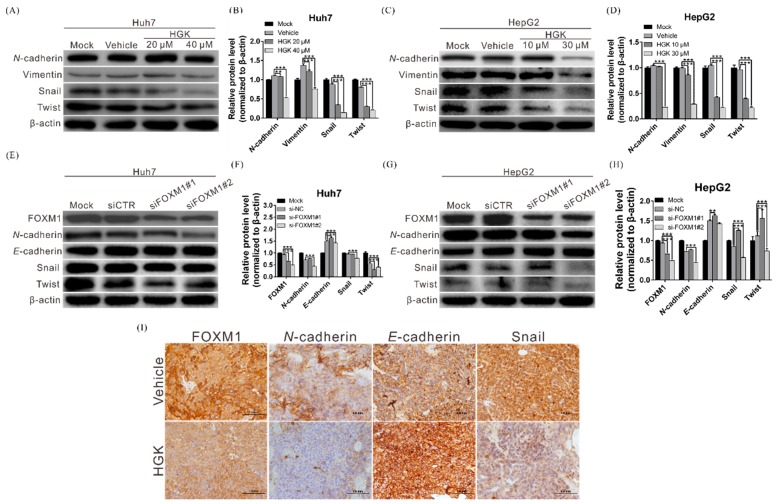
Hydroxygenkwanin (HGK) blocks epithelial–mesenchymal transition (EMT)-related gene expression through the inhibition of FOXM1. (**A**,**C**) Huh7 and HepG2 cells were treated with HGK for 48 h, and the effect of HGK on EMT-related genes were analyzed via western blotting. Quantitative results are shown in (**B**,**D**); ** *p* < 0.01 and *** *p* < 0.001. (**E**,**G**) Western blotting showing the expression of EMT-related proteins in Huh7 and HepG2 cells after silencing FOXM1 expression using small interfering RNA (siFOXM1). Quantitative results are shown in (**F**,**H**). All data were expressed as the mean ± standard deviation values obtained from three independent experiments, ** *p* < 0.01 (**) and *** *p* < 0.001. (**I**) Tumor xenografts from mice treated with HGK or vehicle (DMSO) were dissected and subjected to immunohistochemical staining; the expression of FOXM1 and EMT-related proteins was inhibited in HGK-treated mice.

Previous research has shown that FOXM1 can regulate the expression of EMT-related genes, such as snail, twist, and vimentin, thereby accelerating cell growth and migration [35]. To further elucidate the regulatory relationships among HGK, FOXM1, and EMT, western blotting was performed to investigate HGK activation of FOXM1 and its downstream EMT-related proteins. The expression of EMT-associated proteins in HGK-treated Huh7 and HepG2 cells was significantly reduced (Figure 4A–D). Moreover, silencing the expression of *FOXM1* using siRNA inhibited the expression of the EMT-related proteins (Figure 4E–H). These data indicated that HGK could inhibit the expression of downstream EMT-related genes via its suppression of the transcription factor FOXM1. 

To investigate whether these regulatory relationships also exist in living tissues, immunohistochemical staining was performed on tumor tissues derived from experimental mice to determine the expression of FOXM1 and EMT-related proteins. FOXM1 expression was significantly lower in tissues from HGK-treated mice than in those of their DMSO-administered counterparts (Figure 4I). Additionally, the expression of the EMT-associated genes, namely snail and N-cadherin were also obviously decreased, providing further evidence that HGK suppresses the growth of liver cancer cells by inhibiting the expression of FOXM1 and its downstream EMT-related genes.

### 3.5. HGK Inhibits FOXM1 Expression by Inducing miR-320a Expression

**Figure 5:**
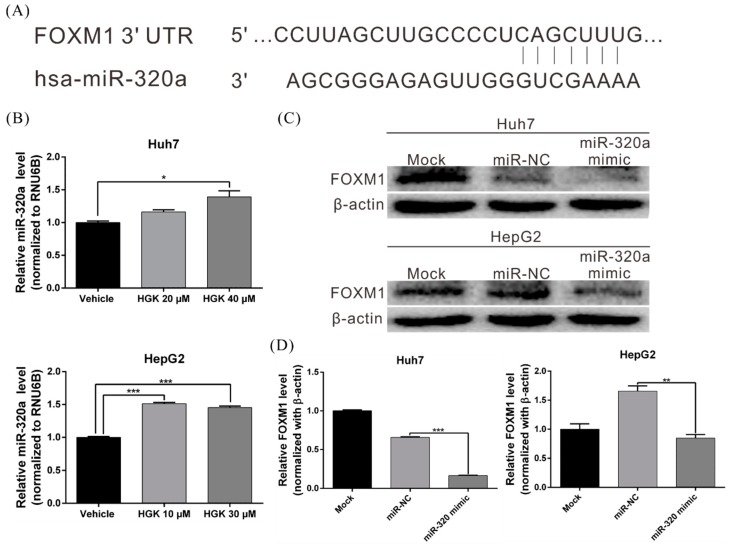
Hydroxygenkwanin (HGK) inhibits FOXM1 expression by inducing miR-320a expression. (**A**) Schematic representation of the predicted miR-320a binding region in the 3′ untranslated region of the FOXM1 mRNA. (**B**) Quantitative reverse transcription PCR showing miR-320a levels after HGK treatment for 48 h; * *p* < 0.05 and *** *p* < 0.001. (**C**) Western blot showing the effect of FOXM1 in Huh7 and HepG2 cells overexpressing an miR-320a mimetic; quantitative results are shown in (**D**). miR-NC: microRNA-negative control. All data are expressed as the means ± standard deviations of three independent experiments.

MiRNAs are one of the main regulators of gene transcription, and previous studies have already shown that FOXM1 expression can be regulated by miR-320a [36,37]. To determine if HGK regulates FOXM1 expression by regulating miR-320a, real-time RT-PCR was used to analyze miR-320a expression in HGK-treated Huh7 and HepG2 cells. HGK was found to induce miR-320a expression by 1.5-fold and 1.6-fold in Huh7 and HepG2 cells, respectively (Figure 5A,B). To further investigate the role of miR-320a in regulating FOXM1, miR-320a mimetics were transfected into Huh7 and HepG2 cells; these mimetics significantly decreased FOXM1 protein level, further indicating that HGK inhibits FOXM1 protein expression in a miR-320a -dependent manner (Figure 5C,D).

**Figure 6:**
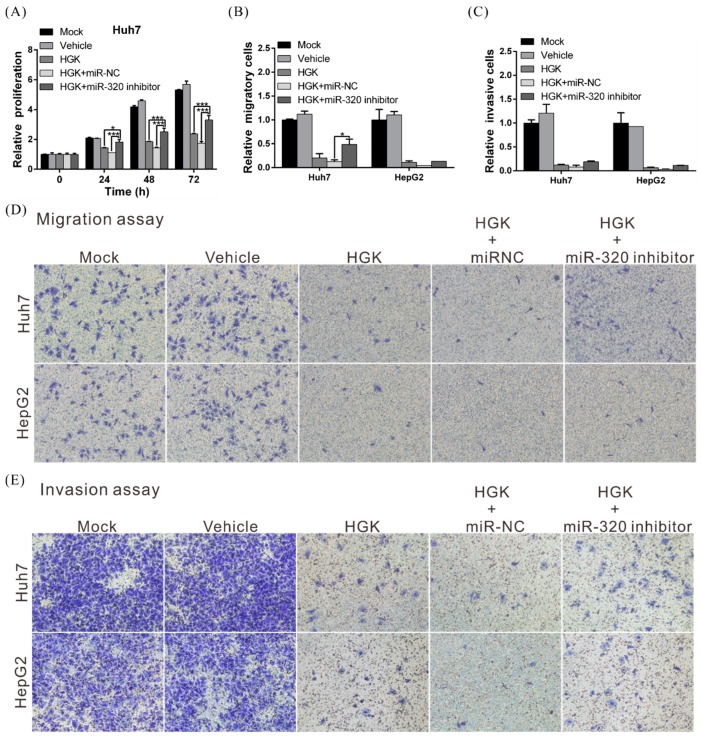
The HGK-mediated suppression of proliferative, migratory and invasive capacities of liver cancer cells is partially rescued by miR-320a inhibitors. The inhibitory effects of HGK on hepatocellular carcinoma cell proliferation (**A**), migration (**B**,**D**), and invasion (**C**,**E**) were reversed following treatment with an miR-320a inhibitor. All data were expressed as the mean ± standard deviation values obtained from three independent experiments; * *p* < 0.05 and *** *p* < 0.001. miR-NC: microRNA-negative control.

To test whether HGK suppresses liver cancer cell aggressiveness via its regulation of the miR-320a/FOXM1/EMT axis, a rescue assay was performed in which HGK-treated cells were exposed to an miR-320 inhibitor. This inhibitor restored cancer cell growth, invasion, and migration in HGK-treated cells (especially Huh7 cells), indicating that the anticancer mechanism of HGK is mediated through miR-320a downregulation of FOXM1 (Figure 6A–E). 

## 4. Discussion

The efficacy of natural products against diseases has been shown in numerous studies, and has led to the use of certain natural products (e.g., artemisinin and curcumin) in current clinical interventions [38,39]. HGK is a flavonoid that is extracted from the flower buds of *D. genkwa*, a commonly used medicinal herb in Southeast Asian countries; however, knowledge of the bioactivity of HGK remains limited to date. In the present study, HGK was found to induce miR-320a expression and inhibit the expression of the transcription factor FOXM1, thereby reducing the expression of FOXM1-regulated EMT-related genes such as N-cadherin, vimentin, twist, and snail. This in turn led to the suppression of the proliferative, migratory, and invasive capacities of liver cancer cells. To the best of our knowledge, the present study is the first to provide evidence of the activity of HGK against liver cancer and its ability to regulate FOXM1 and EMT.

FOXM1 is a key transcription factor in the regulation of cell physiology, and has been shown to modulate the expression of genes associated with the cell cycle and with genome stability [40,41,42]. Previous studies found that FOXM1 dysregulation can cause abnormal cell proliferation, thereby leading to carcinogenesis; its overexpression has been observed in many types of cancer including lung, liver, breast, brain, and oral cancers [43,44,45,46]. Other than serving as a cancer-specific diagnostic marker, FOXM1 is also a key anticancer target [47,48]. The present study revealed that HGK could inhibit both FOXM1 mRNA and protein expression, and also suppressed its downstream signaling pathways. Therefore, HGK can potentially be used in the treatment of other cancers that exhibit FOXM1 overexpression in addition to liver cancer.

The rescue assay showed that the concurrent treatment of liver cells with HGK and an miR-320a inhibitor restored the tumor cells’ proliferative, invasive, and migratory abilities that were initially suppressed by HGK. This indicated that the anticancer activity of HGK is achieved through the regulation of the miR-320a/FOXM1/EMT axis. However, it was also observed that the administration of miR-320a did not lead to a complete reversal of the inhibitory effects of HGK on the liver cancer cells (especially in HepG2 cells), suggesting that other tumor suppression pathways or the expression of other miRNAs may also be regulated by HGK. In agreement with the observation above, the results in Figure 3D–E showed that HGK inhibited the expression of both FOXM1 mRNA and FOXM1 protein, suggesting that HGK may simultaneously regulate FOXM1 expression through two or more mechanisms. As epigenetic regulation is an important and well-known mechanism for regulating gene expression, analysis of the methylation status of FOXM1 gene promoter in HGK-treated cells was also performed. However, no changes were observed compared to the control group (data not shown). As such, further studies are required to determine the detailed mechanisms involved.

The cell function assays revealed that the suppressive effect of HGK was significantly greater in HepG2 cells than in Huh7 cells. The results in Figure 3A also showed that with or without HGK treatment, the gene expression patterns between the Huh7 and HepG2 cells were obviously different, which indicated that in addition to FOXM1, HGK should also inhibit the progression of liver cancer through other regulatory mechanisms. Moreover, as the genetic background of the two cells is different, the pathway of HGK regulation is also altered, resulting in disparate effect of HGK in the two cell lines. However, further research is required to investigate the hypotheses.

In summary, the present study demonstrated that HGK is effective against liver cancer, and that its tumor suppression mechanism is at least partly modulated by its miR-320a-mediated inhibition of both FOXM1 expression and EMT. Moreover, it was observed that HGK has low physiological toxicity. These data may validate EGK as a potential therapeutic agent for the clinical treatment of liver cancer.
